# Analytical evaluation of thirty-two severe acute respiratory syndrome 2 lateral flow antigen tests demonstrates sensitivity remains with the SARS-CoV-2 Gamma lineage

**DOI:** 10.1590/0037-8682-0016-2022

**Published:** 2022-06-06

**Authors:** Konstantina Kontogianni, Daisy Bengey, Dominic Wooding, Kate Buist, Caitlin Greenland-Bews, Christopher Thomas Williams, Margaretha de Vos, Victor Santana Santos, Camille Escadafal, Emily Rebecca Adams, Thomas Edwards, Ana Isabel Cubas-Atienzar

**Affiliations:** 1 Liverpool School of Tropical Medicine, Centre for Drugs and Diagnostics, Liverpool, United Kingdom.; 2 FIND, the global alliance for diagnostics, Geneva, Switzerland.; 3 Universidade Federal de Alagoas, Núcleo de Epidemiologia e Saúde Pública, Arapiraca, AL, Brasil.; 4 Mologic, Bedfordshire, United Kingdom.

**Keywords:** SARS-CoV-2, SARS-CoV-2 Gamma variant, Variant of concern, Diagnostic tests

## Abstract

**Background:**

The emergence of variants of concern (VOCs) requires an ongoing assessment of the performance of antigen lateral flow tests (Ag-RDTs). The limit of detection (LOD) of 32 Ag-RDTs was evaluated using the severe acute respiratory syndrome coronavirus 2 (SARS-CoV-2) Gamma variant.

**Methods:**

Ag-RDTs were performed according to the manufacturer’s instructions with a clinical isolate of the Gamma variant.

**Results:**

Twenty-eight of the 32 Ag-RDTs exceeded the World Health Organization criteria.

**Conclusions:**

This comprehensive analytical evaluation of Ag-RDTs demonstrated that the test performance was maintained with Gamma VOC.

The emergence of the variant of concern (VOC) Gamma (Pango P.1) began in November 2020 in the state of Amazonas, Brazil. The Gamma variant was estimated to be 1.7 to 2.4 times more transmissible than other local strains in Brazil^1^ and quickly started to be detected at increasing rates from January 2021 onwards throughout the country. As a result, this variant has become the predominant lineage associated with the second wave of infections, with over 13 million confirmed cases and 350,000 deaths^2^. 

As of January 10, 2022, the Gamma strain had spread to 93 countries and remained one of the most prevalent variants circulating in the Americas, with the highest frequency in South America. In South America, the proportion of Gamma-associated cases ranges from 5% to 100%, depending on the country. Countries with higher levels of circulation of Gamma variants include Saint Vincent and Grenadines (100%), Haiti (100%), Trinidad and Tobago (50%), Argentina (30%), and Venezuela (30%), followed by Chile, Brazil, Peru, Ecuador, and Suriname, with 10%-20% of the circulating severe acute respiratory syndrome coronavirus 2 (SARS-CoV-2) variants being Gamma^3^.

The use of antigen SARS-CoV-2 lateral flow diagnostic tests (Ag-RDTs) has become one of the first lines of defense against coronavirus disease 2019 (COVID-19)^4^. Ag-RDTs have been shown to be accurate in detecting the vast majority of individuals with a high viral load. In addition, they can determine the presence of SARS-CoV-2 antigens in clinical samples in 10-30 min and have facilitated the early identification and isolation of cases^5^ ^,^ ^6^. In turn, this has slowed transmission, enabled the provision of targeted care, and helped protect health systems^7^.

Gamma has 21 mutations, including 10 in spike (S) and 3 in nucleocapsid (N) proteins. As SARS-CoV-2 Ag-RDTs target S or N proteins, there is a concern that these mutations could affect Ag-RDT performance. Thus, we evaluated the limit of detection (LOD) of 32 commercially available Ag-RDTs using Gamma VOC and compared the results with LODs previously determined with the Alpha (B.1.1.7) and ancestral (B.1) lineages^7^ ^,^ ^8^.

A clinical isolate of the Gamma lineage of SARS-CoV-2 (hCoV-19/Japan/TY7-503/2021) was used for this evaluation. This clinical isolate was obtained from the BEI resources after isolation from a SARS-CoV-2 positive passenger from Brazil in an airport quarantine in Japan in January 2021. The virus stock was propagated into Vero E6 cells (C1008; African green monkey kidney cells), which were maintained at 37 °C with 5% CO_2_ in Dulbecco’s modified Eagle’s medium (DMEM) supplemented with 4.5 g/L glucose and L-glutamine (Lonza, US), 10% fetal bovine serum (Sigma, US), and 50 units/mL of penicillin/streptomycin (Gibco, US).

To determine the LOD of the 32 Ag-RDTs, a fresh aliquot of the third passage of the virus was serially diluted in DMEM from 1.0x10^5^ to 1.0x10^2^ plaque-forming units (pfu)/mL and tested as a direct culture. The viral dilutions were added directly at a 1:10 ratio to the respective Ag-RDT extraction buffers. Ag-RDTs were performed in accordance with the manufacturer’s instructions. Each dilution was tested in triplicate, with Ag-RDT extraction buffer spiked in a 1:10 ratio, with DMEM acting as a negative control. When a 10-fold LOD was found, two-fold dilutions were made and tested to confirm the lowest LOD (LLOD). The LOD was defined as the last dilution of a valid test in which all three replicates were positive. The presence of a control line determined the validity, and only valid tests were included in the analysis. A positive result was interpreted visually by two operators based on the presence of a test line of any intensity. In the event of a discordant result, a third operator read the test and acted as a tiebreaker. All experimental procedures were performed in a containment level 3 laboratory under biosafety level 3 conditions.

Frozen aliquots of the third passage of the virus were quantified by plaque assay as previously described^9^ to determine pfu/mL (gcn/mL). To estimate the genome copy number of each serial dilution, ribonucleic acid (RNA) was extracted using a QIAMP Viral RNA mini kit (Qiagen, Germany), and each viral dilution was tested in triplicate using the COVID-19 Genesig RT-qPCR kit (PrimerDesign, UK). The gcn/mL was calculated from the mean cycle threshold (Ct) values of these replicates. An RNA standard curve was generated by testing five replicates of each 10-fold serial dilution of SARS-CoV-2 synthetic RNA (PrimerDesign, UK). The positive control contained a standard number of copies of the SARS-CoV-2 RNA sequence. Quantitative reverse transcription-polymerase chain reaction (RT-qPCR) was performed using Rotor-Gene Q (Qiagen, Germany).

Once the LOD was determined for each of the 32 Ag-RDTs, the LOD of each Ag-RDT with the Gamma lineage was compared with the ancestral and Alpha SARS-CoV-2 lineages using data from a previous work^5^. Finally, LODs were compared using the Kruskal-Wallis test (IBM SPSS Statistics v28.0). The significance level was set at *P* < 0.05.

We found that 21/32 Ag-RDTs had an analytical LOD ≤ 5.0x10^2^ pfu/mL (ActiveXpress, Bioperfectus, Core Test, Espline, Genedia, Fortress, iChroma, InTec, Joysbio, LumiraDx, Nadal, NowCheck, Panbio, PerkinElmer, RightSign, Roche, Standard F, Standard Q, Strong Step, Sure-Status, and Wantai), fulfilling the acceptable criteria of the British Department of Health and Social Care (DHSC). Additionally, 28/32 (including Biocredit, Covid-go, Excalibur, Mologic, Tigsun, and Wondfo) had a LOD ≤ 1.0x10^6^ gcn/mL, fulfilling the recommendations of the WHO Target Product Profile for SARS-CoV-2 Ag-RDT (Table 1). The most sensitive tests with the Gamma variant were the Core Test and InTec, both of which had analytical LOD of 1.0x10^1^ pfu/mL. The least sensitive tests with the Gamma variant were Innova, Flowflex, Hotgen, Innova, Onsite, and RespiStrip, which had a LOD ≥ 2.5x10^3^ pfu/mL.

**TABLE 1:** Description of the Ag-RDTs evaluated in this study and their limit of detection (LOD) using Gamma as variant of concern

In this Study	Test/Company/Country	Target Ag	LOD (pfu/ml)	LOD (gcn/ml)
ActiveXpress	ActivXpress+ COVID-19 Ag Complete Kit/Edinburgh Genetics Ltd./UK	N	5.0 x 10^2^	2.8 x 10^5^
Biocredit	Biocredit COVID-19 Ag/Rapigen Inc./Rep. Korea	N	1.0 x 10^3^	5.6 x 10^5^
Bioperfectus	SARS-CoV-2 Ag Rapid Test/ Jiangsu Bioperfectus Tech. Ltd./China	N	5.0 x 10^1^	2.2 x 10^4^
Core Test	COVID-19 Ag Test/Core Technology Ltd./China	N	1.0 x 10^1^	3.1 x 10^3^
Covid-Go	Covid-Go/Mologic Ltd./UK	N	1.0 x 10^3^	5.6 x 10^5^
Espline	ESPLINE SARS-CoV-2/Fujirebio Diagnostics Inc./Japan	N	5.0 x 10^2^	2.8 x 10^5^
Excalibur	Rapid SARS-CoV-2 Antigen test card/ Excalibur Healthcare Services/UK	N	1.0 x 10^3^	5.6 x 10^5^
Flowflex	Flowflex SARS-CoV-2 Ag Rapid Test/Acon Biotech, Ltd./China	N	2.5 x 10^3^	1.7 x 10^6^
Fortress	Coronavirus Ag Rapid test cassette/Zhejian Orient Gene Biotech/ China	N	5.0 x 10^2^	2.8 x 10^5^
Genedia	GENEDIA W COVID-19 Ag/ Green Cross Medical Sciences/Rep. Korea	N	5.0 x 10^2^	2.8 x 10^5^
Hotgen	2019-nCoV Antigen test/ Beijin Hotgen Biotech Ltd./China	N	2.5 x 10^3^	1.7 x 10^6^
iChroma	iChroma COVID-19 Ag Test/ Boditech Medical Inc./Rep. Korea	N	1.0 x 10^2^	4.3 x 10^4^
Innova	Innova SARS-CoV-2 Antigen Rapid/ Innova Medical Group Ltd./UK	N	2.5 x 10^3^	1.7 x 10^6^
InTec	Rapid SARS-CoV-2 Antigen test/Intec Products Inc./China	N	1.0 x 10^1^	3.1 x 10^3^
Joysbio	SARS-CoV-2 Antigen Rapid Test Kit/ Joysbio Biotechnology Ltd./China	N	5.0 x 10^2^	2.8 x 10^5^
LumiraDx*	LumiraDx SARS-CoV-2 antigen test/ Lumira Dx Ltd./US	N	1.0 x 10^2^	4.3 x 10^4^
Mologic	Mologic COVID-19 Ag Test device/ Mologic Ltd./UK	N	1.0 x 10^3^	5.6 x 10^5^
Nadal	Nadal COVID-19 Ag Test/Nal von minden GmbH/Germany	N	5.0 x 10^2^	2.8 x 10^5^
NowCheck	NowCheck COVID-19 Ag test/ Bionote Inc./ Rep. Korea	N	1.0 x 10^2^	4.3 x 10^4^
Onsite	Onsite COVID-19 Ag Rapid Test/CTKBiotech Inc./USA	N	5.0 x 10^3^	3.5 x 10^6^
Panbio	Panbio COVID-19 Ag Rapid Test/Abbott Rapid Diagnostics/Rep. Korea	N	1.0 x 10^2^	4.3 x 10^4^
PerkinElmer	PerkinElmer COVID-19 Antigen Test/PerkinElmer/ Switzerland	N	5.0 x 10^2^	2.8 x 10^5^
RespiStrip	Respi-Strip COVID-19 Ag/Coris Bioconcept/Belgium	N	5.0 x 10^3^	3.5 x 10^6^
RighSign	COVID-19 Antigen Rapid Test Cassette/Hangzhou Biotech ltd./China	N	2.5 x 10^1^	1.1 x 10^4^
Roche	SARS-CoV-2 Rapid Ag Test/ Roche Diagnostics/Switzerland	N	5.0 x 10^2^	2.8 x 10^5^
StrongStep	StrongStep SARS-CoV-2 Ag Rapid Test/Nanjing Liming Bio-Products/US	N	5.0 x 10^1^	2.2 x 10^4^
Standard F	Standard F COVID-19 Ag FIA., SD Biosensor Inc./Rep. Korea	N	5.0 x 10^1^	2.2 x 10^4^
Standard Q	Standard Q COVID-19, SD Biosensor Inc./Rep. Korea	N	5.0 x 10^1^	2.2 x 10^4^
Sure-Status	Sure-Status COVID-19 Antigen Card Test, Premier Medical Corp./ India	N	5.0 x 10^2^	2.8 x 10^5^
Tigsun	Tingsun COVID-19 Ag Rapid test/ Beijin Tigsun Diagnostics Ltd./China	N	1.0 x 10^3^	5.6 x 10^5^
Wantai	Rapid SARS-CoV-2 Antigen test/ Wantai Biological Pharmacy Ltd./China	N	1.0 x 10^2^	4.3 x 10^4^
Wondfo	Wondfo 2019-nCoV Antigen Test/ Guangzhou Wondfo Biotech/China	N	1.0 x 10^3^	5.6 x 10^5^

*Microfluidic immunofluorescence technology and no-lateral-flow test.

Of the Ag-RDTs tested with the ancestral lineage, 18/32 had an analytical LOD ≤ 5.0x10^2^ pfu/mL, and 20/32 had a LOD ≤ 1.0x10^6^ gcn/mL. Compared with the Gamma lineage, 15/32 of the Ag-RDTs tested with the ancestral lineage had a lower analytical LOD, 13/32 had a higher LOD, and 4/32 were equal. Of the Ag-RDTs tested with the Alpha lineage, 25/32 Ag-RDTs had an analytical LOD ≤ 5.0x10^2^ pfu/mL, and 30/32 had a LOD ≤ 1.0x10^6^ gcn/mL. Compared with the Gamma lineage, 16/32 Ag-RDTs tested with the Alpha lineage had a lower analytical LOD, 8/32 had a higher LOD, and 8/32 were equal. Six out of 31 Ag-RDTs had a higher sensitivity for the Gamma lineage than the other two lineages (Biocredit, Core Test, Genedia, InTec, Standard F, and Standard Q). Conversely, 5/32 and 9/32 Ag-RDTs showed a higher sensitivity for Alpha and ancestral lineages, respectively, compared with the other two lineages. In total, 12/32 Ag-RDTs met the acceptance criteria for the DHSC and the recommendations of the WHO Target Product Profile for SARS-CoV-2 Ag-RDTs for all three lineages (ActivXpress, Espline, Fortress, iChroma, Joysbio, Lumira, Nadal, NowCheck, Panbio, Roche, Standard Q, and Sure Status). However, there was no significant difference between the Ag-RDT LOD performance with Gamma compared with either the Alpha (Kruskal-Wallis *P* = 0.315) or ancestral lineage (Kruskal-Wallis *P* = 0.378) (Figure 1).

**FIGURE 1:**
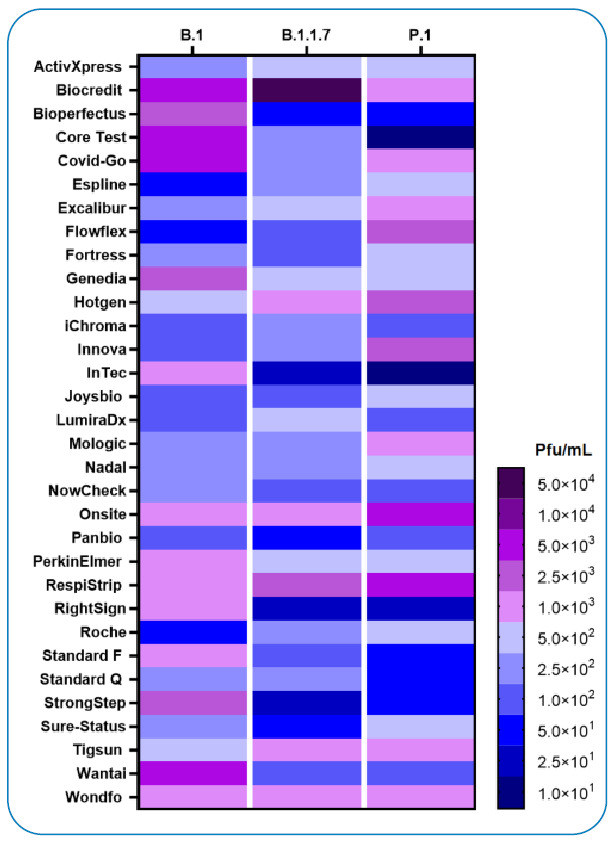
Heatmap comparing the LODs of 32 Ag-RDT using the Gamma (P.1), Ancestral (B.1), and Alpha (B.1.1.7) variants. Data of the Ancestral and Alpha partially taken from our previously published work^4^ ^-^ ^5^. Blue colors indicated LODs fulfilling the DHSC and WHO criteria.

Ag-RDTs have been proven to be essential in our response to the ongoing global SARS-CoV-2 pandemic. Available clinical evaluation data have demonstrated that Ag-RDTs can accurately detect the majority of individuals with high-viral loads^7^. However, most Ag-RDT validation studies were completed prior to the emergence of variants, and to date, there are limited data on the performance of diagnostics using VOCs^10^. As the pandemic progresses, VOCs continue to emerge, outcompete earlier lineages and dominate different areas worldwide. It is important to assess the ability of Ag-RDTs to detect newly emerging variants; therefore, infection prevention and control measures can be updated.

Our results continue to build upon previous work^7^ ^,^ ^8^ ^,^ ^10^ and demonstrate that a selection of commercially available Ag-RDTs can detect the Gamma variant, with some presenting an equivalent performance to the ancestral and Alpha lineages. Despite the observed differences in clinical sensitivities, the Ag-RDTs evaluated here were able, on balance, to detect the ancestral, Alpha, and Gamma lineages.

The Gamma variant has three mutations in the N protein, which appears to be insufficient to disrupt the highly specific antigen-antibody binding reactions that enable the detection of the SARS-CoV-2 antigen. In addition, the N protein has a relatively low mutation rate^7^; therefore, more conserved across different variants. Thus, it has been hypothesized that Ag-RDTs targeting the N protein can detect all known variants^7^. All the Ag-RDTs evaluated in this study targeted the N protein, which contains fewer mutations than the S protein in the Gamma lineage. This may explain the similarities in test performances between the Gamma, Alpha, and ancestral lineages.

Tests targeting the S antigen are expected to have greater difficulty detecting VOCs because of the higher number of mutations in this protein. As Ag-RDTs that target the S protein were not assessed here, we cannot comment on their performance. Further investigations are required to assess the ability of Ag-RDTs to target the S protein to detect VOCs with any sensitivity loss on emerging variants.

Comparing the sensitivities of the Ag-RDTs across different lineages allows continued monitoring of Ag-RDT performance, which could provide early warning signs of a decreased ability to detect VOCs. Twenty-eight out of 32, 20/32, and 30/32 Ag-RDTs met the WHO Target Product Profile for SARS-CoV-2 Ag-RDTs recommendations for the Gamma, Alpha, and ancestral lineages, respectively. This suggests that, despite the comparable performance between different lineages, the differences in LODs have an impact on whether Ag-RDTs satisfy the established criteria. Consequently, some Ag-RDTs may not be suitable for population screening because of their reduced ability to detect certain lineages.

Although the direct application of the cultured virus enabled the evaluation of Ag-RDT analytical sensitivity, it did not replicate the sampling and testing conditions in which the Ag-RDTs were used. Clinical evaluations using patient samples should also be conducted to assess the test performance. Furthermore, the results presented here do not guarantee the ability of the assessed Ag-RDTs to detect other or future VOCs; other strains or variants may contain different N protein mutations that can disrupt detection. As new lineages continue to emerge, there is a need to reassess the ability of Ag-RDTs to diagnose SARS-CoV-2.

One of the limitations of this study is that for practicality, the experiments with the ancestral and Alpha lineages were not performed in parallel to Gamma, and comparative analyses relied on previously published data. Another limitation is that the LOD data were not complemented with accuracy using clinical samples, correlating with Ct values, days of symptom onset, and disease severity, which may interfere with test performance; however, clinical evaluation was outside the scope of this study. 

This is the most comprehensive analytical evaluation of COVID-19 Ag-RDTs with Gamma VOC, and we have demonstrated that the test performance is maintained, indicating Ag-RDT compatibility. This evidence supports their continued usage in countries where Gamma strain is circulating. However, clinical diagnostic evaluations in prospective cohorts from these localities are required to provide definitive data on their clinical performance. Ag-RDTs that target S may have different effects. Diagnostic evaluations must continue to monitor the test performance in emerging variants to ensure continued diagnostic performance.
